# Liver cancer with concomitant *TP53* and *CTNNB1* mutations: a case report

**DOI:** 10.1186/s12907-016-0029-5

**Published:** 2016-06-01

**Authors:** Juliane Friemel, Markus Rechsteiner, Marion Bawohl, Lukas Frick, Beat Müllhaupt, Mickaël Lesurtel, Achim Weber

**Affiliations:** Institute of Surgical Pathology, University and University Hospital Zurich, Schmelzbergstrasse 12, 8091 Zurich, Switzerland; Leibniz Institute for Prevention Research and Epidemiology (BIPS), Bremen, Germany; Clinics of Hepatology and Gastroenterology, University and University Hospital Zurich, Zurich, Switzerland; Swiss Hepato-Pancreato-Biliary Center, Department of Digestive and Transplant Surgery, University Hospital of Zurich, Zurich, Switzerland

**Keywords:** Hepatocellular carcinoma (HCC), Intratumor heterogeneity, *CTNNB1*, *TP53*, Next generation sequencing

## Abstract

**Background:**

In the spectrum of molecular alterations found in hepatocellular carcinoma (HCC), somatic mutations in the WNT/β-catenin pathway and the p53/cell cycle control pathway are among the most frequent ones. It has been suggested that both mutations occur in a mutually exclusive manner and they are used as molecular classifiers in HCC classification proposals.

**Case presentation:**

Here, we report the case of a treatment-naïve mixed hepatocellular/cholangiocellular carcinoma (HCC/CCC) with morphological and genetic intratumor heterogeneity. Within the predominant part of the tumor with hepatocellular differentiation, a p.D32V mutation in exon 3 of the *CTNNB1* gene occurred concomitantly with a *TP53* intron 7/exon 8 splice site mutation*.*

**Conclusion:**

Intratumor heterogeneity challenges the concept of *CTNNB1* and *TP53* gene mutations being mutually exclusive molecular classifiers in HCC, which has implications for HCC classification approaches.

## Background

Hepatocellular carcinoma (HCC) is the fifths most common cancer in men and the second most common cause for cancer-related death worldwide [1]. HCC mostly develop on the background of chronic liver diseases including chronic viral hepatitis due to infection with hepatitis B virus (HBV), or hepatitis C virus (HCV), alcohol-induced liver injury, fatty liver disease or exposure to toxic factors such as aflatoxin. The spectrum of somatic mutations related to liver carcinogenesis has been identified [2]. With marked geographic variation, *TP53* and *CTNNB1* represent two of the most common driver mutations in the African-Asian countries (*TP53*) and in the western world (*CTNNB1*). Several molecular classifications of HCC distinguish HCC with alterations in the p53/cell cycle control pathway from HCCs with alterations in the WNT/β-catenin pathway, including activating mutations of the *CTNNB1* oncogene, *AXIN1* or *APC* [3, 4]. Mutations of *TP53* and *CTNNB1* are largely considered to occur in a mutually exclusive manner [5]. Phenotypical and genetic intratumor heterogeneity with variable mutational status (i.e. wild type among mutated tumor cell clones) of *TP53* and *CTNNB1* in different tumor regions within the same tumor is frequently found in HCC [6]. Here, we describe a *de novo*, hepatitis C-related combined cholangiocellular and hepatocellular carcinoma with marked intratumor heterogeneity on three levels: morphology, immunohistochemical marker profile and mutational status with 3/14 tumor regions of solely hepatocellular differentiation harboring concomitant mutations of *CTNNB1* and *TP53*.

## Case presentation

A liver tumor was detected in a 72 year old male patient with liver cirrhosis Child-Pugh Stage A, a history of type 2 diabetes and chronic hepatitis C virus infection (HCV, genotype 1B), initially diagnosed 13 years ago. Liver enzymes were slightly elevated with alanine aminotransferase 89 U/L (reference: 10–50 U/L) and aspartate aminotransferase 65 U/L (reference: <50 U/L). The 4 cm tumor was detected by routine sonography and removed by laparoscopic liver segment resection.

**Fig. 1:**
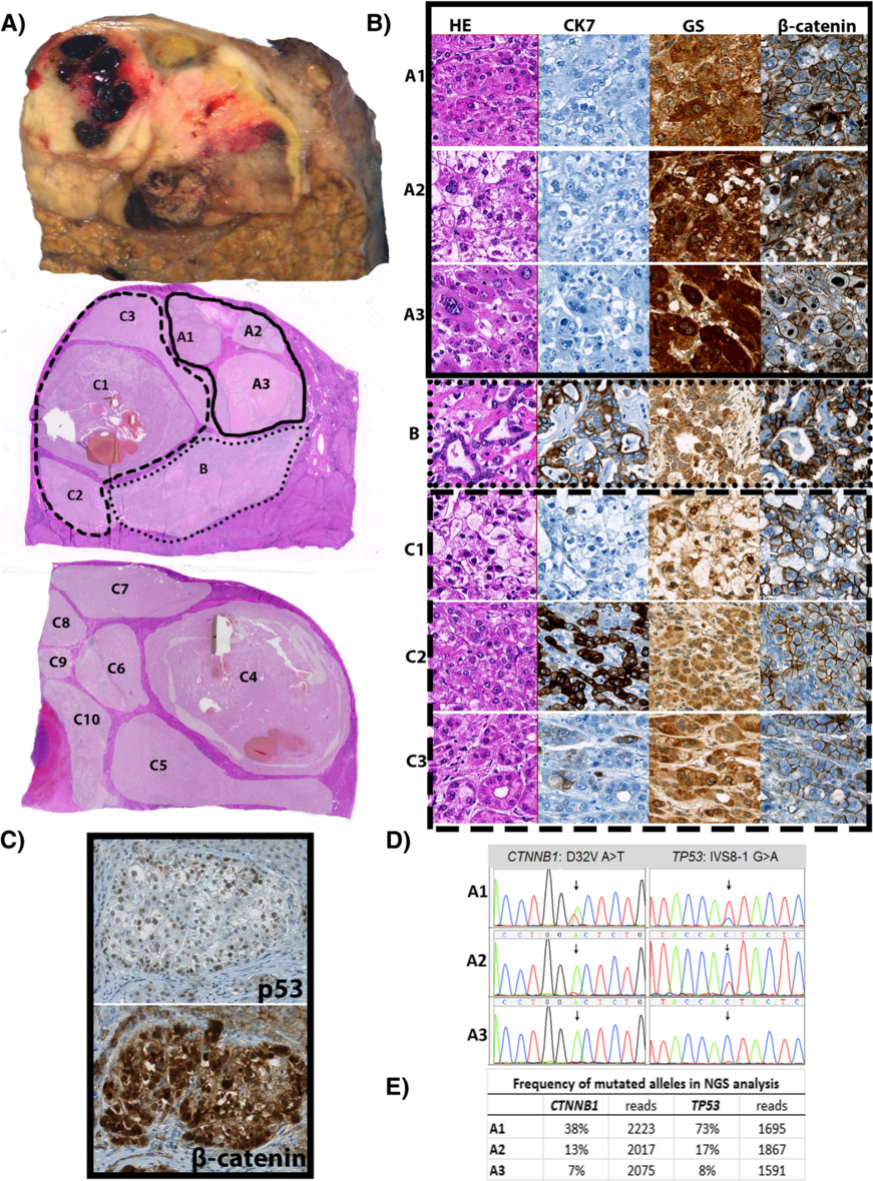
Combined HCC/CCC in a 72 year old male patient with history of hepatitis C and diabetes revealing morphological, immunohistochemical and genetic intratumor heterogeneity. **a** Gross morphology and slide overview (H&E staining) with annotations of defined tumor areas. **b** Microscopic images (40x) of different tumor areas (H&E staining, CK7, glutamine synthetase and β-catenin immunohistochemistry). **c** p53 and β-catenin nuclear positivity in consecutive slides of tumor area A2. **d** Nucleotide exchanges in *CTNNB1* and *TP53* genes (*TP53* depicted as reverse sequence). **e** Mutated allele frequencies of double mutated areas A1-3 with number of gene copies analyzed. Reference sequences: NM 1904.1 (*CTNNB1*) and NM 001126112.2 (*TP53*)

Morphological analysis, immunohistochemistry and multiregional, next generation sequencing (NGS) was applied on representative tumor sections as described [6]. Table 1 and Fig. 1 illustrate histopathological and molecular findings in 14 individual tumor areas, which were grouped into three tumor regions (A, B and C) according to their predominant morphological and molecular characteristics. In summary, a multinodular, combined hepatocellular/cholangiocellular carcinoma, tumor stage T1 grade 2–3, was diagnosed. Intratumoral heterogeneous expression of five liver cell markers (CK7, CK19, glutamine synthetase, p53, β-catenin) was detected including a double positivity for glutamine synthetase, nuclear β-catenin and p53 in tumor region A.

**Table 1:** Morphology, immunohistochemistry and mutational status of individual tumor areas

	Area	A1	A2	A3	B	C1	C2	C3	C4	C5	C6	C7	C8	C9	C10
	Size in mm^2^	7.8	5.54	31.3	65	73.2	16.5	34.5	82.2	20.2	18.9	17.4	28.5	1.94	17.5
Morphology	Solid	+	+	+		+	+	+		+	+		+		
	Glandular (cholangiocellular)				+										
	Trabecular							+	+			+		+	+
	Clear cell change		+			+									+
	Fatty change											+			
	CK19				>50 %		>50 %								
IC	CK7				70 %	SC	50 %	20 %	SC	40 %	SC		SC		50 %
	glutamine synthetase	+	+	+											
	β-catenin nuclear	+	+	+											
	p53	+	+	+											
Mutation	*CTNNB1* sanger seq	D32	D32	wt	wt	wt	wt	wt	wt	wt	wt	wt	wt	wt	wt
	*CTNNB1* deep seq	D32	D32	D32^a^	wt	wt	wt	wt	wt	wt	wt	wt	wt	wt	wt
	*TP53* sanger seq	IVS8-1	IVS8-1	wt	wt	wt	wt	wt	wt	wt	wt	wt	wt	wt	wt
	*TP53* deep seq	IVS8-1	IVS8-1	IVS8-1^a^	wt	wt	wt	wt	wt	wt	wt	wt	wt	wt	wt

*sanger seq* sanger sequencing, *deep seq* deep sequencing (NGS), *wt* wild type, *SC* single cells positive

^a^low frequency (<10 %)

Next generation sequencing was performed with a minimum coverage of 1329 (SD ± 725) reads per amplicon of every single tumor area. Sequencing results yielded a p.D32V (c.363 A > T) mutation and a *TP53* ivs8-1 (c.783-1 G > A) splice site mutation in tumor region A. Comparing mutated allele frequencies, *CTNNB1* and *TP53* gene copies showed a similar range of both frequencies in area A1-3 (Fig. 1). All mutated and wild type tumor areas additionally displayed a SNP of exon 7 (rs17880604). Collectively, morphological and immunohistochemical findings together with sequencing results demonstrated that a tumor subclone with hepatocellular differentiation had concomitant *CTNNB1* and *TP53* gene mutations.

To date, after a follow-up time of 12 months, the patient had a local recurrence of a liver tumor which was inoperable and therefore treated by transarterial chemoembolization (TACE).

## Conclusion

In this case of a HCV infection -related liver cancer, a *CTNNB1/TP53* double mutation was detected in a tumor region of hepatocellular differentiation, among *TP53* and *CTNNB1* wild type tumor areas. The analysis of mutated allele frequencies using next generation sequencing techniques corroborates that the double mutation is located in the same tumor cell population. To our knowledge, this is the first detailed description of a *CTNNB1/TP53* double mutation in a single liver cancer lesion. *TP53* and *CTNNB1* both are molecular classifiers for hepatocellular carcinoma. For instance, in the transcriptome-analysis based classification proposal by Boyault et al. [4], six HCC subgroups are distinguished: two groups are characterized by *TP53* and two independent groups by *CTNNB1* alterations. A study by Laurent-Puig et al. [5] on genetic alterations in hepatocarcinogenesis describes *TP53* and *CTNNB1* mutations as mutually exclusive. In agreement, a study by Tornesello et al. [7] records mutations of the two driver genes as being mutually exclusive.

*CTNNB1* mutations are reported to be associated with hepatitis C infections [8]. *TP53* point mutations frequently are reported to occur specifically at codon 249 after aflatoxin exposition. The frequency and the causal link between *TP53* and *CTNNB1* mutations in HCC have not been systematically investigated. A study by Ötztürk et al. [9] on HCC cell lines provides evidence that inactivation of *TP53* could cause aberrant nuclear β-catenin accumulation, suggesting a link between the two genes. In the presented case study, the *CTNNB1* mutation affected the GSK-3β phosphorylation site [10] which argues for a β-catenin accumulation independent from the *TP53* mutation. The detected *TP53* mutation affects a splice site of exon 8. Although splice sites in *TP53* are not typical mutation sites, there is evidence that *TP53* splicing mutations lead to exon dropping indicating biological relevance [11]. Furthermore, the nuclear accumulation of the dysfunctional p53 protein in immunohistochemical analysis found in this case supports a functional significance of this splice site mutation. As has been reported, wild type p53 is rapidly degraded while mutations lead to nuclear accumulation of the p53 protein [12, 13].

In summary, the molecular, immunohistochemical and morphological diversity in the presented case indicates a high level of intratumor heterogeneity and challenges the concept that *TP53* and *CTNNB1* are mutually exclusive driver alterations in HCC. Distinct parts of the tumor reveal multinodularity, and differ with respect to their biomarker expression and mutational status, indicative of distinct tumor subpopulations [14]. This finding illustrates the challenge to molecularly characterize individual HCC. Routine pathological analysis is based on testing a small piece of a tumor, assuming that it represents the whole tumor. When analyzing distinct pathways of hepatocarcinogenesis such as the WNT/β-catenin pathway as a potential therapeutic target in HCC [15], it will be pivotal in the future to also take into account the level of intratumor heterogeneity.

## Abbreviations

CCC, cholangiocellular carcinoma; CTNNB1, Catenin β 1; HCC, hepatocellular carcinoma; TACE, transarterial chemoembolization; TP53, tumor protein 53
